# A Point-Matching Method of Moment with Sparse Bayesian Learning Applied and Evaluated in Dynamic Lung Electrical Impedance Tomography

**DOI:** 10.3390/bioengineering8120191

**Published:** 2021-11-25

**Authors:** Christos Dimas, Vassilis Alimisis, Nikolaos Uzunoglu, Paul P. Sotiriadis

**Affiliations:** Department of Electrical and Computer Engineering, National Technical University of Athens, 15780 Athens, Greece

**Keywords:** electrical impedance tomography, method of moment, sparse Bayesian learning, inverse problem, lung imaging, image reconstruction

## Abstract

Dynamic lung imaging is a major application of Electrical Impedance Tomography (EIT) due to EIT’s exceptional temporal resolution, low cost and absence of radiation. EIT however lacks in spatial resolution and the image reconstruction is very sensitive to mismatches between the actual object’s and the reconstruction domain’s geometries, as well as to the signal noise. The non-linear nature of the reconstruction problem may also be a concern, since the lungs’ significant conductivity changes due to inhalation and exhalation. In this paper, a recently introduced method of moment is combined with a sparse Bayesian learning approach to address the non-linearity issue, provide robustness to the reconstruction problem and reduce image artefacts. To evaluate the proposed methodology, we construct three CT-based time-variant 3D thoracic structures including the basic thoracic tissues and considering 5 different breath states from end-expiration to end-inspiration. The Graz consensus reconstruction algorithm for EIT (GREIT), the correlation coefficient (CC), the root mean square error (RMSE) and the full-reference (FR) metrics are applied for the image quality assessment. Qualitative and quantitative comparison with traditional and more advanced reconstruction techniques reveals that the proposed method shows improved performance in the majority of cases and metrics. Finally, the approach is applied to single-breath online in-vivo data to qualitatively verify its applicability.

## 1. Introduction

Electrical impedance tomography (EIT) is a medical imaging technique which reveals the conductivity or admittance distribution of a subject under test (SUT) [1]. In EIT, an alternating, low-amplitude current usually up to 1MHz is induced into a cluster of electrodes, while the measured electrode potentials are used as raw data for the image reconstruction. Unlike other medical imaging modalities, EIT is characterized by the absence of ionizing radiation, its low cost and its notable temporal resolution. This makes EIT a useful tool for real-time lung function monitoring. Many studies have shown the importance of EIT for revealing vital signs related to ventilation properties, such as tidal volume (TV) [2], or pathological situations, such as acute respiratory distress syndrome (ARDS) [3].

Despite its potential advantages, EIT is lacking in spatial resolution, something that still keeps its application in medical equipment limited. In addition, EIT images often present artefacts that, in some cases, may degrade their clinical value and diagnostic efficacy. Such artefacts are related to the highly ill-posed and ill-conditioned nature of the EIT inverse reconstruction problem. This means that the image quality presents with high sensitivity to voltage signal noise. In real-time EIT imaging, the signal-to-noise ratio (SNR) is often limited, because higher frequency currents need to be injected in order to achieve a high temporal resolution [4,5]. Indeed, in practice, lower frame rate EIT hardware systems [6,7] usually present better voltage SNR levels than higher frame rate systems [8,9].

Furthermore, EIT is susceptible to modeling errors. Particularly in dynamic thoracic imaging, the lack of a homogeneous background, the  patient’s unknown chest boundary shape, as well as its changes over time due to the patient’s breathing cycles and the high chance of the electrodes’ displacement during signal acquisition also introduce significant modeling errors [10,11,12]. Despite the fact that a percentage of modeling errors can be compensated when time-difference EIT is applied, their impact on image quality may be still noticeable. Moreover, EIT is a highly non-linear problem, which means that highly inhomogeneous admittance inclusions cannot be accurately estimated with simple linear methods [12]. The variations in the lungs’ admittance due to the continuous change in air volume is a typical case [13].

Over the years, many approaches have been proposed for EIT image reconstruction. The most simple approaches assume small conductivity or admittance inclusions and linearize the problem around a predefined homogeneous value. Then, the problem is treated directly using either truncated singular value decomposition (TSVD) or standard Tikhonov regularization (STR), where an identity matrix prior is considered. Generalized Tikhonov regularization (GTR) schemes [14] such as the Laplace prior, the NOSER prior [15] and the high-pass filter Gaussian prior [16] can be also applied, improving the image reconstruction performance compared to TSVD and STR. To compensate boundary movement effects, [17,18] introduced electrode movement priors, combining them with the previous schemes. Another single-step approach which makes use of a figure-of-merit (FoM) framework to optimize both parameters and performance is the Graz consensus reconstruction algorithm for EIT (GREIT) [19]. GREIT uses dual-mesh schemes, i.e., a coarse 2D domain for the admittance reconstruction and a fine 3D domain, extruded from the coarse 2D one, for accurate forward calculations. This greatly improves accuracy, but at the cost of additional time and complexity.

Despite the benefits of single-step approaches in dynamic EIT imaging speed, EIT is a non-linear problem. Therefore, most linear approaches cannot accurately capture significant conductivity changes. In such cases, iterative approaches have been developed to deal with the non-linearity. A common iterative framework is based on the Gauss–Newton (GN) algorithm, which makes use of the GTR schemes mentioned above ($L^{2}$-norm regularization) [20]. Another popular iterative approach is the total variation (TV) approach, where $L^{1}$-norm regularization priors are used [21,22,23]. A hybrid non-linear difference EIT imaging approach was proposed in [12,24] in an effort to effectively deal with both the models’ mismatches and the problem’s non-linearity. Another $L^{1}$-norm approach uses the Bregman distance scheme, showing improved performance in lung imaging compared to the traditional TV scheme [25].

Sparse Bayesian learning (SBL) was firstly proposed as a mathematical formulation in [26,27,28] but was only recently applied in EIT [29,30,31,32]. Instead of the traditional regularization schemes, it treats the inverse problem as a log-likelihood optimization procedure, assuming a sparse conductivity distribution. SBL approaches show robustness to signal noise, while the non-trivial hyperparameter selection needed in regularization techniques is avoided. Although some SBL methods, such as structure-aware SBL (SA-SBL) [30] and time-sequence learning SBL [33] have been proposed for EIT, their evaluation is limited to simple circular and cylindrical structures. Hence, SBL has not been applied in dynamic thoracic imaging, where the structures have more complex geometries, usually unknown, and are highly non-homogeneous.

The point-matching method of moment (PM-MoM) for EIT was also recently proposed in [34]. It uses a global integral equation approach with Green’s functions. The logarithm of conductivity is expressed as a linear combination of modified radial basis functions (RBFs). Contrary to the traditional finite element (F.E.) approach, which treats the problem as a weak form that does not hold for significant conductivity changes, the PM-MoM is formulated globally, decreasing the problem’s non-linearity. The PM-MoM has therefore shown to converge faster than the traditional F.E. approaches both in $L^{1}$ and $L^{2}$-norm inverse problem schemes. Despite the fact that PM-MoM has been tested on circular and cylindrical structures, it has not been quantitatively evaluated in dynamic thoracic imaging.

Motivated by the benefits of the PM-MoM and the SBL optimization scheme, in this work we introduce an approach that combines these two methods. In particular, the proposed PM-MoM SBL approach undertakes the image reconstruction problem’s non-linearity, offering robustness to noise and reduced susceptibility in modeling errors. To evaluate the PM-MoM SBL approach, we apply it to 3D F.E. thoracic structures (cases) based on 3 male subjects’ CT images, available online. For each case, five sub-structures are built, considering five corresponding breath-cycle states from expiration to the end-inspiration. Each structure includes the lungs, heart, vertebrae, muscle and skin tissues to avoid the assumption of a uniform background [11]. It is noted that most previous studies for EIT approaches in dynamic thoracic imaging consider only the inspiration and expiration ends and only the lung tissues in their models for quantitative evaluation. The proposed approach is compared with traditional (GN, TV, difference of absolute images) and more advanced (prior movement, hybrid non-linear imaging) F.E.-based regularization approahces, as well as the regularized MoM approach, showing increased noise robustness and improved performance both qualitatively and quantitatively. Finally, the proposed method is tested in in vivo human breath data which is available online, verifying its proper applicability.

The rest of this paper is organized as follows. In Section 2, the EIT problem’s principle, as well as state-of-the-art regularization-based methods, are outlined. In Section 3, the proposed PM-MoM SBL approach is presented, while in Section 4, the 3D thoracic structures, the evaluation FoM, the method adopted to extract the reference images and the in vivo data are described. In Section 5, the image reconstruction results, as well as the quantitative results, are demonstrated and discussed. Finally, Section 6 concludes this work.

## 2. Background

In this section, a brief review of the EIT mathematical formulation and the state-of-the-art inverse reconstruction approaches used for the comparisons is performed.

### 2.1. EIT Principle

Assume a *N*-electrode EIT setup and a *d*-dimensional domain (d∈{2,3}) Ω, where the current is injected. The problem can be described according to the following Laplace equation:

(1) $$\nabla \left(\sigma (r)\nabla u(r)\right)=0,\;r\in \Omega$$

that implies the following boundary conditions, according to the complete electrode model (CEM) [35]:

(2) $$\begin{array}{cc} u\left(r\right)+z_{l}\sigma \left(r\right)\frac{\partial u(r)}{\partial n} & =U_{l},\;r\in e_{l},\;l=1,...,N \end{array}$$

(3) $$\begin{array}{cc} \int_{e_{l}}\sigma \left(r\right)\frac{\partial u(r)}{\partial n}dS & =I_{l},\;l=1,...,N \end{array}$$

(4) $$\begin{array}{cc} \sigma \left(r\right)\frac{\partial u(r)}{\partial n} & =0,\;r\in \partial \Omega \backslash {⋃}_{l=1}^{N}e_{l} \end{array}$$

where $r\in R^{d}$ is the position vector, σ(r) is the conductivity, u(r) is the potential, n is the normal outward-pointing vector, $e_{l}$ is the electrodes’ positions set, $z_{l}$ is the electrodes’ contact impedances, $U_{l}$ is the *l*th electrode voltage and $I_{l}$ is the current injected on the *l*th electrode.

### 2.2. Time-Difference EIT

In time-difference (dynamic) EIT imaging, we assume two consecutive states. The corresponding computed boundary voltages’ vectors can be written as follows:

(5) $$U^{\left(1\right)}=\left[U_{1}^{1}\;U_{2}^{1}\;...\;U_{Nm}^{1}\right]\in R^{Nm\times 1}$$

and

(6) $$U^{\left(2\right)}=\left[U_{1}^{2}\;U_{2}^{2}\;...\;U_{Nm}^{2}\right]\in R^{Nm\times 1},$$

where *m* is the total voltage measurements acquired for each current injection electrode pair, according to the selected measurement pattern [36]. Accordingly, we assume that two voltage data measurement frames $V^{\left(1\right)}\in R^{Nm\times 1}$ and $V^{\left(2\right)}\in R^{Nm\times 1}$ are acquired from the EIT system. We then set the differential voltage frames

(7) $$\delta U=U^{\left(2\right)}-U^{\left(1\right)}$$

and

(8) $$\delta V=V^{\left(2\right)}-V^{\left(1\right)}.$$

Considering Gaussian noise $e_{n}\in R^{Nm\times 1}$ between the simulated boundary voltages and the measurements, we have

(9) $$\delta V=\delta U+e_{n}.$$

Furthermore, we assume $\sigma^{\left(1\right)}\left(r\right)$ and $\sigma^{\left(2\right)}\left(r\right)$ as the corresponding conductivity distributions, setting the difference

(10) $$\delta \sigma \left(r\right)=\sigma^{\left(2\right)}\left(r\right)-\sigma^{\left(1\right)}\left(r\right).$$

If the finite element method (F.E.M.) discretization scheme is applied in Ω, we assume a number of *L* elements and that each element *i* presents a constant conductivity $\sigma_{i}$.

Hence, we can write the following conductivity vectors:

(11) $$\sigma^{\left(1\right)}={\left[\sigma_{i}^{\left(1\right)}\right]}_{i=1}^{L}\in R^{L\times 1}$$

(12) $$\sigma^{\left(2\right)}={\left[\sigma_{i}^{\left(2\right)}\right]}_{i=1}^{L}\in R^{L\times 1}$$

(13) $$\delta \sigma ={\left[\delta \sigma_{i}\right]}_{i=1}^{L}\in R^{L\times 1}.$$

The general approach is to formulate the inverse problem as a weighted-least squares (WLS) minimization problem between δV and δU, adding a regularization term P(δσ) to stabilize the problem’s ill-conditioned nature, such that

(14) $$F\left(\delta \sigma \right)={∥\delta U-\delta V∥}_{W}^{2}+\lambda^{2}P\left(\delta \sigma \right).$$

(15) $$\delta \sigma_{*}=\underset{\delta \sigma \in R^{L}}{\mathrm{argmin}}\left\{F(\delta \sigma )\right\},$$

where $W\in R^{Nm\times Nm}$ is a diagonal, noise covariance matrix and λ is the regularization hyperparameter. The problem is to find the optimal δσ that minimizes F(δσ) in (14). From hereon, we assume that all the measurement channels have the same noise; hence, $W=I_{\mathrm{Nm}}$.

### 2.3. Single-Step Linear Reconstruction

As mentioned in the introduction, EIT image reconstruction is a non-linear problem, i.e., the relation between U and σ(r) is non-linear. However, assuming relatively small conductivity changes, and using Taylor approximation around a linearization point $\sigma_{o}$, we can write

(16) $$\delta U=\frac{\partial \delta U}{\partial \delta \sigma }{|}_{\sigma_{o}}\delta \sigma +O\left({∥\delta \sigma ∥}^{2}\right)≃J\delta \sigma ,$$

where $J\in R^{Nm\times L}$ is the Jacobian matrix around $\sigma_{o}$. In the simplest case (dimensionless electrodes), J is computed according to the following formula [37]:

(17) $$J_{dm}^{i}=\frac{\partial \delta U_{dm}}{\partial \delta \sigma }=-\int_{\Omega_{i}}\nabla u\left(I^{d}\right)\cdot \nabla u\left(I^{m}\right)dA,$$

where $\Omega_{i}$ denotes the *i*-th element’s domain, *d* denotes the current injection differential channel and *m* denotes the voltage measurement differential channel. The minimization function is then written according to the following form:

(18) $$F\left(\delta \sigma \right)={∥J\delta \sigma -\delta V∥}_{W}^{2}+\lambda^{2}P\left(\delta \sigma \right).$$

In this case, smooth $L^{2}$ priors, such as the standard Tikhonov, the Laplace, the NOSER or the Gaussian priors, are commonly utilized [14,15,16]. Hence, we can write

(19) $$P\left(\delta \sigma \right)={∥\delta \sigma ∥}_{Q}^{2},$$

where $Q\in R^{L\times L}$ is the prior matrix. The linearized problem has the following closed-form solution:

(20) $$\delta \sigma_{*}={\left(J^{T}WJ+\lambda^{2}Q\right)}^{-1}J^{T}W\delta V.$$

This is the most commonly used linear EIT scheme.

### 2.4. Regularized Reconstruction Approaches

In this section, we perform a brief review of the regularization-based linear and non-linear approaches used for the comparisons.

(I) *Non-Linear Gauss–Newton (GN):* The traditional non-linear GN approach makes an iterative estimation of conductivity change distribution to optimize

(21) $$F\left(\delta \sigma \right)={∥\delta U-\delta V∥}_{W}^{2}+\lambda^{2}{∥\delta \sigma ∥}_{Q}^{2}.$$

An initial solution is taken from (20). Then, J, as well as δσ, are re-estimated in each iteration until convergence.

(II) *Total Variation (TV):* This non-linear, iterative approach assumes that intense conductivity changes occur between neighbouring elements of Ω by applying $L^{1}$-norm priors. The functional to be minimized is written as follows:

(22) $$F\left(\delta \sigma \right)={∥\delta U-\delta V∥}_{W}^{2}+\lambda^{2}\sum_{i=1}^{N_{ed}}\sqrt{{∥L_{i}\delta \sigma ∥}^{2}+\beta },$$

where $N_{ed}$ is the total number of edges between the domain’s elements, and $L\in R^{N_{ed}\times L}$ is a sparse matrix which shows the relation between the elements and their edges. $L_{i}$ refers to the *i*th row of the matrix L, and β>0 is a parameter that prevents the non-differentiability of the regularization term [22]. In terms of this paper, the primal-dual interior point (PD-IPM) TV method is adopted [21,22,23].

(III) *Movement Prior:* This approach, which was firstly proposed by [17] and furtherly developed in [18], performs linear difference-EIT reconstruction while considering the electrodes’ movement effect. The electrode movement $\delta x\in R^{dN\times 1}$ is also estimated along with the conductivity change. To this end, Tikhonov and Laplace priors have been proposed for the simultaneous estimation of δσ and δx, properly modifying the Jacobian matrix J and the prior matrix Q [17,18]. Apart from λ, a μ>0 regularization hyperparameter for the electrode movement prior is needed. In terms of this paper, both δσ and δx estimations are performed using the Laplace prior, in the way described in [18]. This approach has proven to reduce the artefacts caused by the electrodes’ movement and boundary changes and has been exclusively developed for applications in dynamic lung imaging.

(IV) *Difference of Absolute Images:* In this approach, absolute, instead of difference, EIT reconstruction is applied particularly for each measurement frame [38]. The problem’s objective functions are the following:

(23) $$F_{1}\left(\sigma^{\left(1\right)}\right)={∥U^{\left(1\right)}-V^{\left(1\right)}∥}_{W}^{2}+\lambda^{2}{∥\sigma^{\left(1\right)}∥}_{Q}^{2}.$$

and

(24) $$F_{2}\left(\sigma^{\left(2\right)}\right)={∥U^{\left(2\right)}-V^{\left(2\right)}∥}_{W}^{2}+\lambda^{2}{∥\sigma^{\left(2\right)}∥}_{Q}^{2}.$$

Defining $\sigma_{*}^{\left(1\right)}\in R^{L}$ and $\sigma_{*}^{\left(2\right)}\in R^{L}$ as the obtained solutions from (23) and (24), respectively, the final estimated conductivity change is simply obtained by

(25) $$\delta \sigma_{*}=\sigma_{*}^{\left(2\right)}-\sigma_{*}^{\left(1\right)}$$

In this paper’s reconstructions, the minimization of $F_{1}$ and $F_{2}$ is performed by using the absolute GN non-linear approach with a Laplace prior.

(V) *Multiple Priors (Non-Linear Difference Imaging—N.L.D.):* This approach, proposed in [12,24], concatenates the voltage data measurement frames as follows:

(26) $$\bar{V}={\left[{V^{\left(1\right)}}^{T}\;{V^{\left(2\right)}}^{T}\right]}^{T}\in R^{2Nm\times 1},$$

and the computed boundary voltages as follows:

(27) $$\bar{U}={\left[{U^{\left(1\right)}}^{T}\;{U^{\left(2\right)}}^{T}\right]}^{T}\in R^{2Nm\times 1},$$

while it is assumed that conductivity changes occur in a particular region of interest ($\Omega_{ROI}\subseteq \Omega$), which is discretized in $L_{ROI}\leq L$ elements, such that

(28) $$\sigma^{\left(2\right)}=\sigma^{\left(1\right)}+M\delta \sigma_{\mathrm{ROI}},$$

where M is an operator that maps $\delta \sigma_{\mathrm{ROI}}$ with the domain’s elements. The following optimization problem is defined:

(29) $$F\left(\bar{\sigma }\right)={∥\bar{U}-\bar{V}∥}_{W}^{2}+\lambda_{1}^{2}{∥\sigma^{\left(1\right)}∥}_{Q_{1}}^{2}+\lambda_{2}^{2}\sum_{i=1}^{N_{ed,ROI}}\sqrt{{∥L_{i,\mathrm{ROI}}\delta \sigma_{\mathrm{ROI}}∥}^{2}+\beta },$$

where $\lambda_{1}$ and $\lambda_{2}$ are regularization hyperparameters, $N_{ed,ROI}$ is the total number of edges between the ROI elements and $L_{i,\mathrm{ROI}}\in R^{N_{ed,ROI}\times L_{ROI}}$ shows the relation between the ROI’s elements and their edges. A smooth $L^{2}$-norm prior (prior matrix $Q_{1}$) is used to estimate $\sigma^{\left(1\right)}$, while a $L^{1}$-norm prior is used to estimate the local conductivity change $\delta \sigma_{\mathrm{ROI}}$. The solution ${\bar{\sigma }}_{*}$ that minimizes (29) is achieved via an iterational process. A linesearch to perform the updates is essential due to the problem’s high non-linearity.

It is worthwhile to mention that apart from these methods, many other approaches have been developed for difference-EIT imaging. For example, the TSVD, a traditional linear approach, gives similar results to (20) by performing thresholding SVD instead of regularization. Finally, a very well-known single-step approach is the D-Bar, which uses a non-linear scattering transform and low-pass filtering [39,40,41]. A comparison between D-Bar and regularized methods can be found in [42].

## 3. Method-of-Moment with Sparse Bayesian Learning (Pm-Mom SBL)

This section presents the proposed method as a combination of the PM-MoM system matrix formulation and an SBL approach for the occurring inverse problem.

The PM-MoM formulates a global integral equation (holding in the whole Ω) instead of using the weak form of (1). It expresses the voltages and fields as Green’s functions and their gradients, respectively. The conductivity is non-linearized in the integral equation, and, unlike the conventional F.E.M. formulation, is not assumed to be a piecewise constant at each element. Instead, its logarithm is expressed as a summary of radial basis functions (RBFs) [34].

The governing integral equation takes the following form:

(30) $$u\left(r;r_{+},r_{-}\right)=\int_{\Omega }G\left(r,r^{\prime }\right)\nabla \left(\ln\sigma (r^{\prime })\right)\cdot \nabla u_{o}\left(r^{\prime };r_{+},r_{-}\right)dA+u_{o}\left(r;r_{+},r_{-}\right),$$

where $G(r,r^{\prime })$ denotes the Green’s function set between an observation point r and a source point $r^{\prime }$, $u_{o}$ denotes the domain’s voltage distribution when the conductivity is constant (homogeneous with a value $\sigma_{o}$) and $r_{+}$, $r_{-}$ denote the electrode coordinates from where the current is sourced and sinked, respectively. It is also found that

(31) $$u_{o}\left(r^{\prime };r_{+},r_{-}\right)=\frac{I}{\sigma_{o}}\left[G\left(r,r_{+}\right)-G\left(r,r_{-}\right)\right].$$

We then express the logarithm of conductivity as follows:

(32) $$\ln\sigma (r^{\prime })=\ln\left(\sigma_{o}\right)+\sum_{j=1}^{L}c_{j}\theta \left(r^{\prime },r_{J}\right),$$

where $r_{J}$ is the *j*th pixel’s center point. The RBF θ is selected according to the following generalized form:

(33) $$\theta \left(r^{\prime },r_{J}\right)=exp\left(-\frac{{∥r^{\prime }-r_{J}∥}_{p_{1}}^{p_{1}/p_{2}}}{2D^{2}}\right),$$

where $p_{1}>0$ and $p_{2}>0$ are integers ($p_{1}$ even), and *D* is a parameter which adjusts the RBF’s width. By discretizing (30), replacing the conductivity logarithm with (32) and taking the electrodes voltages’ differences, according to the measurement pattern adopted and the method described in [34], we are led to a linear system of equations

(34) $$M^{o}c=\delta U,$$

where $M^{o}\in R^{Nm\times L}$ is the system matrix, $c={\left[c_{j}\right]}_{j=1}^{L}\in R^{L\times 1}$ is the weighting coefficients’ vector and $\delta U\in R^{Nm\times 1}$ denotes the numerically expected electrode potentials’ differences. Adopting the measurement model described by (9), we treat the inverse problem as a minimization of the following WLS objective function:

(35) $$F\left(c\right)={∥M^{o}c-\delta V∥}_{W}^{2}+\lambda^{2}P\left(c\right).$$

The minimization of (35) can be performed with the traditional $L^{2}$ or $L^{1}$-norm regularized approaches [34]. Unlike J, the matrix $M^{o}$ occurs directly from the discretization of (30) without the assumption of $\ln\left(\sigma \left(r^{\prime }\right)\right)≃\sigma \left(r^{\prime }\right)-1$ near $\sigma_{o}$. Hence, the expression of the boundary voltages as a function of conductivity is more accurate, leading to a faster convergence of the inverse solution. We note that the Green’s function *G* and its gradient can be separately precomputed either analytically for canonical geometries or by using the F.E.M. or the finite difference method (F.D.M.) (at the same discretization mesh as MoM) to solve the Laplace equation for the potential and the field for non-canonical geometries.

In this particular work, we make use of an SBL formulation [30] to minimize (35). To this point, we interpret the objective function in a Bayes log-likelihood context

(36) $$F_{B}\left(c\right)=\ln p\left(\delta V|c\right)+\lambda \ln p\left(c;\Theta \right),$$

where Θ is a set of hyperparameters. Considering that c is a superposition of some clusters overlapping each other with an equal size *h*, and g=L−h+1 is the total number of clusters, the following factorization is performed:

(37) $$c=\Psi x=\left[\Psi_{1},...,\Psi_{g}\right]{\left[{x_{1}}^{T},...,{x_{g}}^{T}\right]}^{T},$$

where $x_{i}\in R^{h\times 1}$ and $\Psi_{i}={\left[0_{(i-1)\times h}^{T}I_{h\times h}0_{(L-i-h+1)\times h}^{T}\right]}^{T}\in R^{L\times h}$. The Gaussian noise model (9) is approximated as

(38) $$\delta V=M^{o}c+e_{n}=M^{o}\Psi x+e_{n}.$$

We also define $\Phi =M^{o}\Psi \in R^{Nm\times gh}$. Furthermore, we assume that the weight vector $x\in R^{gh\times 1}$ obeys the following Gaussian distribution:

(39) $$p\left(x;{\left\{\gamma_{i},B_{i}\right\}}_{i=1}^{g}\right)=N\left(0,\Sigma_{0}\right)$$

with zero mean value and $\Sigma_{0}\in R^{gh\times gh}$ covariance matrix.

Considering the hyperparameters $\Theta =\{\gamma_{o},{\left\{\gamma_{i},B_{i}\right\}}_{i=1}^{g}\}$ and adopting the expectation-minimization (EM) method, as in [30], we get an a posteriori estimation of the weight vector’s x mean values vector $\mu_{x}\in R^{gh\times 1}$ and covariance matrix $\Sigma_{x}\in R^{gh\times gh}$. Hence, we get a maximum a posteriori (MAP) estimation of x. For clarity, we summarize the SBL process in Algorithm 1.

The updating rule for $\gamma_{i}$ is based on the majoration-minimization method [26,43]. In addition, instead of the linearized Jacobian matrix, we use the PM-MoM $M^{o}$ system matrix as an input in order to limit the problem’s non-linearity effect and avoid the necessity of recalculating J.

**Figure 1:**
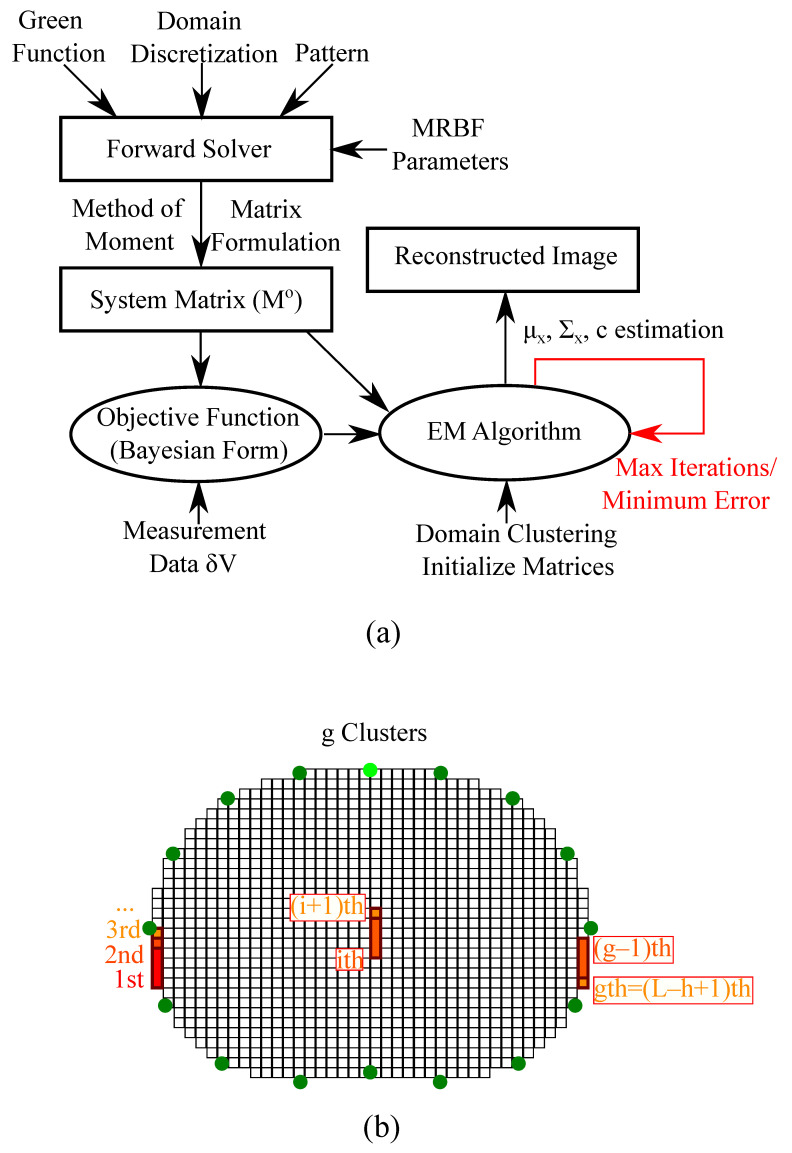
(**a**) Simplified demonstration of the PM-MoM SBL method process. (**b**) Illustrative example of a thoracic pixelized domain’s clustering structure.

The SBL method shows increased robustness to noise and modeling errors, while the reconstruction artefacts are minimized. Furthermore, unlike the traditional regularized schemes, the choice of the hyperparameter *h* (number of clusters) slightly affects the reconstruction quality [30]. Hence, the cumbersome and non-trivial process of hyperparameter selection is avoided. Moreover, the regular “moment” grids used in PM-MoM are suitable for performing sparse-based reconstructions. However, despite recent developments, the SBL methods are overall characterized by high complexity. For instance, the SBL approach applied presents a complexity of $O(N^{2}m^{2}gh)$ per iteration. Nevertheless, avoiding the search process for optimal hyperparameters partially reduces the increased complexity effects. A simplified flow chart of the whole PM-MoM SBL, as well as an example of a domain’s clustering, are depicted in Figure 1. The SBL approach adopted in this particular paper is in the form used in [30]. However, some modified SBL approaches using approximate message passing (AMP) to accelerate the E-step of the algorithm have been researched for 3D imaging [31], frequency-difference EIT [32] and multiple measurement vector (MMV) time-sequence measurements [33]. Such techniques can also be appropriately combined with the PM-MoM in a similar manner.
**Algorithm 1:** Sparse Bayesian learning (SBL).**Inputs:**$M^{o}$, δV, *h*, $\epsilon_{min}$, $i_{max}$**Initialize:**ϵ=1, κ=0, $\mu_{x}=0_{gh\times 1}$, $\Sigma_{x}=0_{\mathrm{gh}\times \mathrm{gh}}$, $\gamma =\mathrm{diag}\left(I_{g\times g}\right)\in R^{g\times 1}$,$\gamma_{o}=0.01\times \sqrt{\frac{1}{Nm-1}\sum_{j=1}^{Nm}{\left|\delta V_{j}-\bar{\delta V}\right|}^{2}}$, $B_{i}=\mathrm{Toeplitz}\left(\left[1,\zeta^{1},...,\zeta^{h-1}\right]\right)$, ζ=0.9, Ψ,$\Sigma_{0}=\left[\begin{array}{ccc} \gamma_{1}B_{1}\; & ...\; & 0_{h\times h}\\ ...\; & ...\; & ...\\ 0_{h\times h}\; & ...\; & \gamma_{g}B_{g} \end{array}\right]$, $\Phi =M^{o}\Psi$, $\Sigma_{u}=\gamma_{o}I_{Nm\times Nm}+\Phi \Sigma_{o}\Phi^{T}$, $\tilde{B_{i}}=B_{i}$.**LOOP:****While** $\epsilon >\epsilon_{min}$ and $\kappa \leq \kappa_{max}$ **do**1.      $\mu_{x}:=\Sigma_{o}\Phi^{T}\Sigma_{u}^{-1}$2.      $\Sigma_{x}:=\Sigma_{0}-\Sigma_{0}\Phi^{T}\Sigma_{u}^{-1}\Phi \Sigma_{0}$3.      $\gamma_{o}:=\frac{1}{Nm}\left({∥\delta V-\Phi \mu_{x}∥}_{2}^{2}+\sum_{i=1}^{g}\mathrm{tr}\left(\Sigma_{x}^{i}\Phi_{i}^{T}\Phi_{i}\right)\right)$4.      $\gamma_{i}:=\gamma_{i}\cdot \frac{{∥\sqrt{B_{i}}\Phi_{i}^{T}\Sigma_{u}^{-1}\delta V∥}_{2}}{\sqrt{\mathrm{tr}\left(\Phi_{i}^{T}\Sigma_{u}^{-1}\Phi_{i}B_{i}\right)}}$,    for each cluster i∈{1,...,g}.5.       $\tilde{B_{i}}:=\tilde{B_{i}}+\frac{1}{\gamma_{i}}\left({\Sigma_{x}}^{i}+{\mu_{x}}^{i}{\left({\mu_{x}}^{i}\right)}^{T}\right)$,    for each cluster i∈{1,...,g}.6.       ${\tilde{r}}_{i}:=\frac{\bar{\mathrm{diag}\left(\tilde{B_{i}},1\right)}}{\bar{\mathrm{diag}\left(\tilde{B_{i}}\right)}}$,    for each cluster i∈{1,...,g}.7.       $r_{i}:=\mathrm{sign}\left({\tilde{r}}_{i}\right)\cdot \min\left\{\left|{\tilde{r}}_{i}\right|,0.99\right\}$,    for each cluster i∈{1,...,g}.8.       $B_{i}:=\mathrm{Toeplitz}\left(\left[r_{i}^{0},...,r_{i}^{h-1}\right]\right)$,    for each cluster i∈{1,...,g}.9.       Update $\Sigma_{0}$ and $\Sigma_{u}$.10.       $\epsilon =\frac{{∥\mu_{x}^{new}-\mu_{x}^{prev}∥}_{2}}{{∥\mu_{x}^{new}∥}_{2}}$11.       κ:=κ+1**End****Output:** $c_{*}=\Psi \mu_{x}$Estimate σ(r) using (32) and (33).

## 4. Evaluation Methods

In this section, the thoracic structures’ extraction and the corresponding tissues’ electrical properties are demonstrated. In addition, the evaluation metrics, including GREIT FoMs with minor modifications, the Pearson CC, the RMSE and the recently proposed FR are briefly explained. Finally, an in vivo online available dataset demonstrating a subject’s full-breath cycle is briefly described.

### 4.1. Thoracic Structures

To examine and compare the previously described algorithms’ performance in dynamic imaging, we have created 3 3D fine F.E. thoracic structures based on 3 CT-images of 3 corresponding different healthy adult male subjects. The CT images were taken between the third and the fourth intercostal levels and are included in a large medical database which is available online in [44]. The 3D models have been created in MATLAB using the EIDORS and the NETGEN software [45,46] and include the following tissues: left lung, right lung, heart, vertebra and skin, while muscle is assumed to be the background.

For each structure, 5 breath-cycle states have been considered from end-expiration (deflated) to end-inspiration (inflated). Hence, a total number of 15 F.E. models have been created, demonstrating 3 subjects’ cases in 5 breath-cycle states. Each state presents chest boundary changes (a total change of 5–8% of the chest’s width) and lung shape changes (total expansion 10–15% of the lungs’ width at the inflated state) [12]. The lungs’ admittance changes between the states have also been considered.

The tissues’ admittance values are loaded from an open-source database, demonstrated in [47,48,49]. All admittance values depend on the selected current signal frequency *f* in which the EIT measurements are performed, while the lungs’ admittances also depend on the breathing state. For this particular work, we assume f=100 kHz, which is in the range of the current frequencies used for dynamic lung imaging. This is actually a common frequency choice for such applications [7,50,51]. Nevertheless, higher frequencies, such as those applied in high-performance modern EIT systems [7,9], can be also considered. Furthermore, to take into account each tissue’s inhomogeneity, the following standard deviations (std) of the admittance values assigned to each tissues’ elements have been taken into account: 1% for the skin, 2% for the heart and the muscle background and 3% for both lungs. These values fall within the range depicted in the mentioned database.

The conductivity and permittivity values assigned to each tissue at 100 kHz, as well as their std are shown in Table 1. For the lungs, the deflated and inflated states’ values were taken from [47,48,49]. To find the intermediate states’ values, we firstly assumed that the lungs’ volumes increase linearly over time during the inhalation process [52]. A relative (arbitrary unit—A.U.) volume has been defined as follows:

(40) $$F_{i}=\frac{3}{P+1}i+\frac{3}{P-1}+4,\;\mathrm{for}\;1\leq i\leq P,$$

where *P* is the total number of states from end-expiration to the end-inspiration. In our case, P=5. In actuality, the lungs’ volume change is more complex and heavily depends on each particular breath. The main changes in the lungs’ admittance occur due to the air-flow. The lungs’ conductivity as a function of their volume can be expressed by [52,53]

(41) $$\sigma_{l}=K_{1}\left(\frac{0.85s_{b}}{w}+0.03s_{i}\right)\frac{32F+4.5}{{\left(32F+9\right)}^{2}}+K_{2},$$

where $s_{b}$, *w* and $s_{i}$ are lung morphological parameters described in [53], with their values selected to be $s_{b}=0.5$, w=1.5 and $s_{i}=2$. In addition, $K_{1}$ and $K_{2}$ are coefficients used to scale the lungs’ conductivity between the known values and end-inspiration and end-expiration. The permittivity of the lungs is correspondingly defined as [53]

(42) $$\epsilon_{l}=L_{1}\left(\frac{0.85e_{rb}}{w}+780F^{1/3}e_{rm}\right)\frac{32F+4.5}{{\left(32F+9\right)}^{2}}+L_{2},$$

where $e_{rb}$ and $e_{rm}$ are also lung morphological parameters described in [53], with their values selected at $e_{rb}=10^{4}$ and 10, respectively. Furthermore, $L_{1}$ and $L_{2}$ are scaling coefficients. We note that blood-cycle related changes have not been taken into consideration, since the HR frequency is 3–6 times higher than the breath frequency.

In each case (1–3), the boundary extracted from the corresponding CT image is used as a cross-section to create the 3D F.E. structure. For each structure, a height of h=1 A.U. has been considered, while the x-axis limits have been normalized between −1 and 1 A.U. A number of N=16 circular electrodes of radius $R_{el}=0.05$ A.U. have been placed at the z=1/2 A.U. level. In addition, an electrode position error has been added: 5% height std and 3% angle std, since this is a more realistic case.

**Figure 2:**
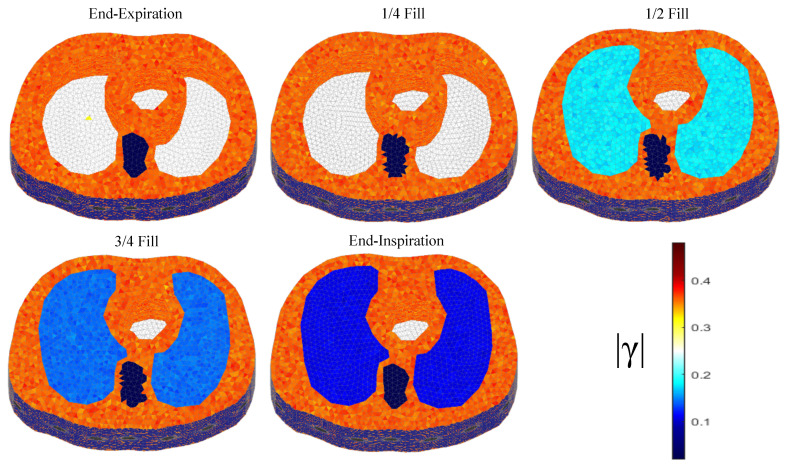
The 3D F.E. structure for the 1st subject case in 5 breath cycle states.

**Figure 3:**
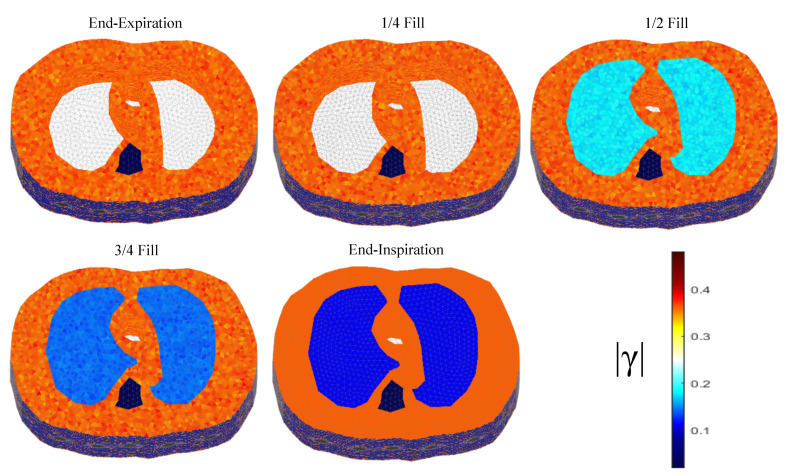
The 3D structure for the 2nd subject case in 5 breath cycle states.

**Figure 4:**
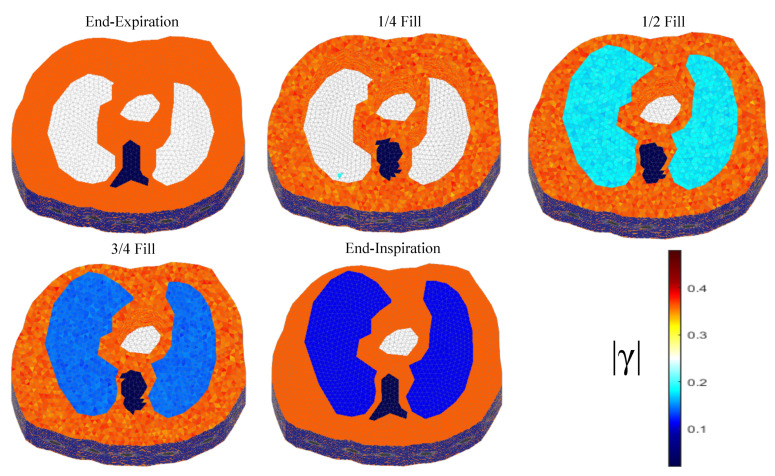
The 3D structure for the 3rd subject case in 5 breath cycle states.

**Figure 5:**
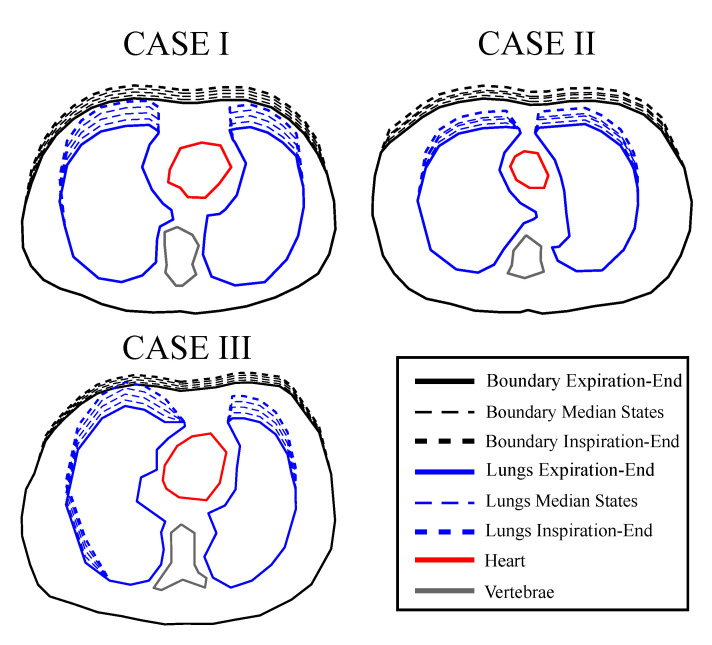
Cross-sectional boundary and lung shape changes from the end-expiration to the end-inspiration states. The extracted shapes were used to create extruded 3D models using NETGEN.

The thoracic structures are demonstrated in Figure 2, Figure 3 and Figure 4. Their boundary and lungs’ shape changes are demonstrated at the cross-section level in Figure 5. Finally, the numbers of each model’s tetrahedral elements and nodes are shown in Table 2.

To simulate the measurements, the adjacent (skip-0) current and voltage measurement pattern was considered [36,54], while a Gaussian noise of −50dB SNR was added to the extracted raw signals. The EIDORS library tool in MATLAB was used to perform the simulations [45].

### 4.2. Reconstruction Domain

When the EIT image reconstruction is performed using simulated models, we need to avoid *inverse crime.* This occurs when the simulated model’s and the reconstruction domain’s mesh or boundary is equal [55]. Instead, the reconstruction needs to be performed on a significantly different mesh, usually coarser than the simulated model’s one.

**Figure 6:**
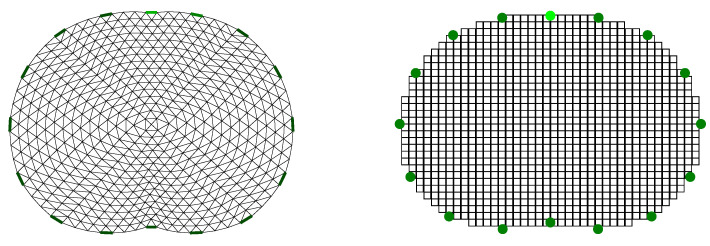
Reconstruction domain Ω used for the EIT imaging. **Left**: F.E. mesh. **Right**: MoM mesh.

In this work, all the image reconstructions are performed on a 2D coarse thoracic-shaped domain, called Ω, which presents a different boundary than any of the original model’s boundaries. For the FEM-based reconstruction approaches, the domain contains L=1024 triangular elements and $n_{e}=545$ nodes. The shunt electrode model has been assumed to simulate the electrodes’ effects [35,56]. Furthermore, for the PM-MoM reconstructions, a L=1060 uniform pixel grid has been used, considering the electrodes as points [57]. The reconstruction domain for the two discretizations is shown in Figure 6.

### 4.3. Reference Image Extraction

In order to perform a quantitative evaluation of EIT imaging, a corresponding “ground truth” reference image has to be defined on the reconstruction domain Ω. However, the simulated models have completely different shapes and discretization meshes compared to Ω in both the standard FEM and MoM reconstruction cases. In order to “match” the simulated models with the reconstruction domain, the approach presented in [58] is adopted.

This approach considers that the simulated models’ shape in all three cases is not constant, as well as that difference-EIT imaging is performed. Hence, we firstly get five absolute reference images, each one representing a particular state. Then we take the differences between the 2nd–5th images and the 1st “reference frame”, resulting in 4 reference images.

Each “true boundary” domain ${\tilde{\Omega }}_{k,l}$, k={1,2,3}, l={1,2,...,5} is extracted from the 3D models’ electrodes’ cross-section plane. Then, we scale Ω and each ${\tilde{\Omega }}_{i,j}$ in the *x*-axis by normalizing its limits between −1 and 1. We secondly define $A_{i}$ as the Ω *i*th-element’s/pixel’s area, with i={1,2,...,L}. Then, the percentage of $A_{i}$ which is within the curves defined by the following six tissues, left lung, right lung, vertebra, heart, skin and muscle, is expressed as a weight vector,

(43) $$w_{k,l}^{i}={\left[w_{j,k,l}^{i}\right]}_{j=1}^{6}\in R_{+}^{6\times 1},$$

for the *k*th case and *l*th state. At this point, we define the vector

(44) $$\gamma_{t,l}={\left[\gamma_{j,l}\right]}_{j=1}^{6}\in C^{1\times 6},$$

which represents the mean admittances for each one of the 6 mentioned tissues at the *l*th state. Then, the *i*th element’s or pixel’s reference admittance is estimated as follows:

(45) $$\gamma_{r,k,l,i}=\gamma_{t,l}\cdot w_{k,l}^{i}\in C.$$

The *k*th case, *l*th state (absolute) reference admittance vector is then defined as

(46) $$\gamma_{r,k,l}={\left[\gamma_{r,k,l,i}\right]}_{i=1}^{L}\in C^{L\times 1}.$$

For each image frame, we get the following difference reference admittance vector:

(47) $$\delta \gamma_{r,k,l+1}=\gamma_{r,k,l+1}-\gamma_{r,k,1},$$

where $\delta \gamma_{r,k,l+1}\in C^{L\times 1}$ for l={1,2,3,4}, assuming the *k*th case.

**Figure 7:**
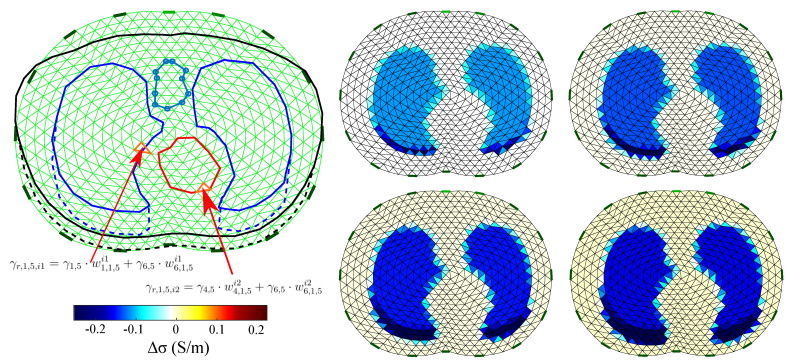
Visual example of the reference images’ extraction for the 1st thoracic case (k=1). **Left**: example of the absolute admittance extraction of the $i_{1}$th and $i_{2}$th elements when in an inflated case (l=5). **Right**: the difference image reference frames for case k=1 when F.E.M. is applied. Only the conductivity difference (real values) is expressed in the legend.

A simple example of this process and the F.E.M. reference images for k=1 are demonstrated in Figure 7.

### 4.4. Figures of Merit

To quantitatively evaluate the EIT reconstructions, we use the following five GREIT FoM: target amplitude—TA, position error—PE, shape deformation—SD, resolution—RES and ringing—RNG [19]. These have been properly adapted to the examined thoracic cases. Furthermore, the CC, the RMSE and the FR metrics are applied.

#### 4.4.1. Target Amplitude—TA

Assume $\delta \sigma_{*}\in R^{L\times 1}$ isthe conductivity difference estimated from the EIT reconstructions. The TA can be defined as the normalized summary of elements’/pixels’ amplitudes in the image,

(48) $$TA=\frac{\sum_{i=1}^{L}\delta \sigma_{*,i}}{\max\left\{|\delta \sigma_{*,i}|\right\}}.$$

TA is a FoM similar to the amplitude response—AR, which is considered to be the most important GREIT FoM [19]. Its absolute value should be relatively low and stable during the breath process. When admittance values are reconstructed, TA can be estimated by taking only the real values.

#### 4.4.2. Position Error—PE

The position error—PE—shows the precision of the reconstructed inclusions’ center of gravity. In our case, we define the right and the left lung as the corresponding inclusions. Then, we get two PE values:

(49) $$PE_{LL}=\left|r_{\mathrm{tLL}}-r_{\mathrm{iLL}}\right|,$$

for the left lung, where $r_{\mathrm{tLL}}$ is the true center of the left lung and $r_{\mathrm{iLL}}$ is the reconstucted left lung’s center, and

(50) $$PE_{RL}=\left|r_{\mathrm{tRL}}-r_{\mathrm{iRL}}\right|$$

for the right lung, where $r_{\mathrm{tRL}}$ is the true center of the right lung, and $r_{\mathrm{iRL}}$ is the reconstucted right lung’s center. The total PE is given by

(51) $$PE=PE_{LL}+PE_{RL}.$$

To detect an inclusion as “lung”, we first filter the reconstructed image, setting all the elements’/pixels’ absolute values that are below a selected threshold (−1/4 of the maximum absolute value) to zero and all non-zero values to 1. We denote $x_{f}\in R^{L\times 1}$ as the filtered image conductivity distribution, where

(52) $$x_{f_{i}}=\left\{\begin{array}{cc} 1 & \mathrm{if}\;\delta \sigma_{*,i}\leq -1/4\max\left\{|\delta \sigma_{*}|\right\}\\ 0 & \mathrm{otherwise} \end{array}\right.$$

**Figure 8:**
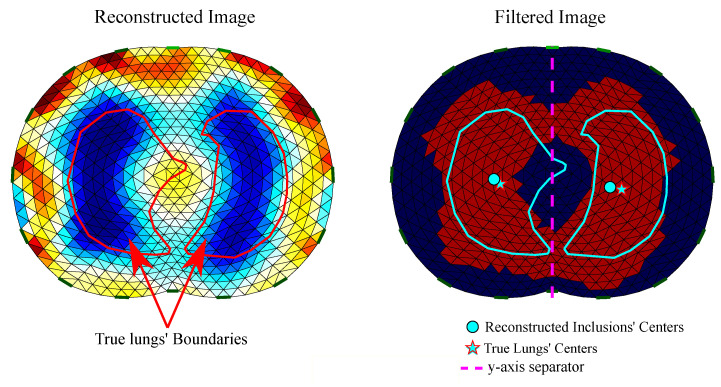
Reconstructed EIT image filtering and detection of PE. **Left**: raw reconstructed image including the true lungs’ boundaries (for the corresponding 3D model’s cross-section). **Right**: filtered image including true and reconstructed lungs’ centers.

Secondly, the left and right lung inclusions are separated in the reconstructed image with a *y*-axis line, as shown in Figure 8.

#### 4.4.3. Shape Deformation—SD

Shape deformation—SD—denotes the percentage of the reconstructed and filtered inclusion which is not within the “true lung’s” boundary. Assume LL and RL are the “true” left and right lungs’ domains, respectively. If $x_{f}$ is the filtered reconstructed image conductivity distribution, we get

(53) $$SD_{LL}=\frac{\sum_{i\notin LL}x_{f_{i,left}}A_{i}}{\sum_{i\in LL}x_{f_{i,left}}A_{i}}$$

for the left lung and for each element/pixel left from the *y*-axis with an area $A_{i}$ (see Figure 8). For the right lung we have

(54) $$SD_{RL}=\frac{\sum_{i\notin RL}x_{f_{i,right}}A_{i}}{\sum_{i\in RL}x_{f_{i,right}}A_{i}}$$

for each element/pixel right from the *y*-axis with an area $A_{i}$.

The total SD is given by

(55) $$SD=\frac{\sum_{i\notin LL}x_{f_{i,left}}A_{i}+\sum_{i\notin RL}x_{f_{i,right}}A_{i}}{\sum_{i\in LL}x_{f_{i,left}}A_{i}+\sum_{i\in RL}x_{f_{i,right}}A_{i}}.$$

SD should also be low and stable.

#### 4.4.4. Resolution—RES

If $A_{LL}$ represents the reconstructed left lung inclusion’s area, $A_{RL}$ is the reconstructed right lung inclusion’s area and $A_{o}$ is the Ω area, the resolution—RES—is given by

(56) $$RES=\sqrt{\frac{A_{LL}+A_{RL}}{A_{o}}}.$$

We can estimate $A_{LL}$ and $A_{RL}$ from the following expressions

(57) $$A_{LL}=\sum_{i\in LL}x_{f_{i,left}}A_{i}$$

and

(58) $$A_{RL}=\sum_{i\in RL}x_{f_{i,right}}A_{i},$$

RES should be low and uniform [19].

#### 4.4.5. Ringing—RNG

Ringing—RNG—demonstrates whether the reconstructed inclusion causes areas of opposite sign near the target inclusion. It is given by

(59) $$RNG=\frac{\sum_{i\notin LL\&i\notin RL\&\delta \sigma_{*,i}<0}\delta \sigma_{*,i}}{\sum_{i\in LL}\delta \sigma_{i}+\sum_{i\in RL}\delta \sigma_{*,i}}.$$

RNG is an important GREIT FoM, since in dynamic lung image reconstructions, conductive areas often appear between the lungs that are sometimes wrongly recognized as “heart” [19]. It should also be low and as stable as possible.

Apart from the above GREIT parameters, we also apply the following FoM.

#### 4.4.6. Pearson Correlation Coefficient—CC

The Pearson correlation coefficient—CC—is one of the most common metrics that quantify an image’s quality. It indicates the similarity between a “ground truth” reference image and the reconstructed image. It is given by

(60) $$CC=\frac{\mathrm{Cov}(\delta \sigma_{*},\delta \sigma_{r})}{\mathrm{std}\left(\delta \sigma_{*}\right)\mathrm{std}\left(\delta \sigma_{r}\right)},$$

where $\delta \sigma_{r}=Re\left\{\delta \gamma_{r}\right\}$ for each particular case *k* and state *l*.

#### 4.4.7. Root Mean Square Error—RMSE

An additional FoM used for the image evaluation is the well-known RMSE, which is estimated according to the following formula:

(61) $$RMSE=\sqrt{\frac{\sum_{i=1}^{L}{\left(\delta \sigma_{r,i}-\delta \sigma_{*,i}\right)}^{2}}{L}}.$$

#### 4.4.8. Full Reference—FR

This metric was recently proposed as a universal FoM for EIT systems’ evaluation on the reconstructed images [59]. It has been extensively presented and applied in phantom experimental setups. However, this is the first time that FR is applied in dynamic thoracic models.

To estimate FR, the normalization of the reference images $\delta \sigma_{r}$ and the reconstructed images $\delta \sigma_{*}$ (element/pixel) data between −1 and 1 needs to be performed. We define as $\mathrm{EDref}\in R^{L\times 1}$ the normalized reference image data and $\mathrm{EDtest}\in R^{L\times 1}$ the normalized reconstructed image data.The global FR (GFR) is defined as follows:

(62) $$GFR=0.5\cdot \sum_{i=1}^{L}\left|EDref_{i}-EDtest_{i}\right|.$$

We also define the local FRs for the left and the right lungs, respectively, as

(63) $$FR_{LL}=0.5\cdot \sum_{i\in LL}\left|EDref_{i}-EDtest_{i}\right|$$

and

(64) $$FR_{RL}=0.5\cdot \sum_{i\in RL}\left|EDref_{i}-EDtest_{i}\right|$$

Both local and global FR indicate a high-quality reconstruction when they take low values.

### 4.5. In Vivo Data

A qualitative comparison is attempted using online available in vivo EIT data [45]. This data consists of 34 data frames of a single breath cycle captured by the 16-electrode serial-data EIT Scanner [60] using the adjacent current and voltage measurement pattern. This system performs demodulation of the input signal with an AM signal of a higher order of magnitude frequency than that of the electrode voltage signal. The injected current frequency (carrier) signal was set at 65 kHz. Since difference-EIT imaging is performed, the first frame is used as reference, resulting in 33 image reconstructions.

## 5. Results and Discussion

Image reconstructions were performed for the 3 structures presented in Section 4.1, considering each one of the 5 mentioned breathing states and resulting 4 images per structure. Reconstructions were also performed for the in vivo data described in Section 4.5, resulting in 33 EIT images. The regularization scheme-based approaches described in Section 2.4, the MoM-regularized approach, as well as the proposed MoM SBL approach described in Section 3 were used. Particularly for the multiple priors difference non-linear approach (N.L.D.), we consider that the ROI where $\delta \sigma_{ROI}$ occurs is equal to Ω, since the “lungs” area covers a significant part of Ω [12].

For all cases, the reconstruction hyperparameter λ value, as well as the μ, β and *h* parameters’ values (for the movement-prior, TV and PM-MoM SBL reconstructions, respectively) were heuristically selected, as shown in Table 3. The selection of λ was performed in such a way that CC is maximized. Although some methods for the λ selection, such as the L-curve, the noise figure (NF) and the BestRes calibration methods [19,61], have been proposed, this process is beyond this work’s scope. Finally, for the PM-MoM SBL, we set the maximum number of iterations $\kappa_{max}$ to 5 and the minimum tolerance $\epsilon_{min}$ to $10^{-5}$.

### 5.1. Simulation Results

**Figure 9:**
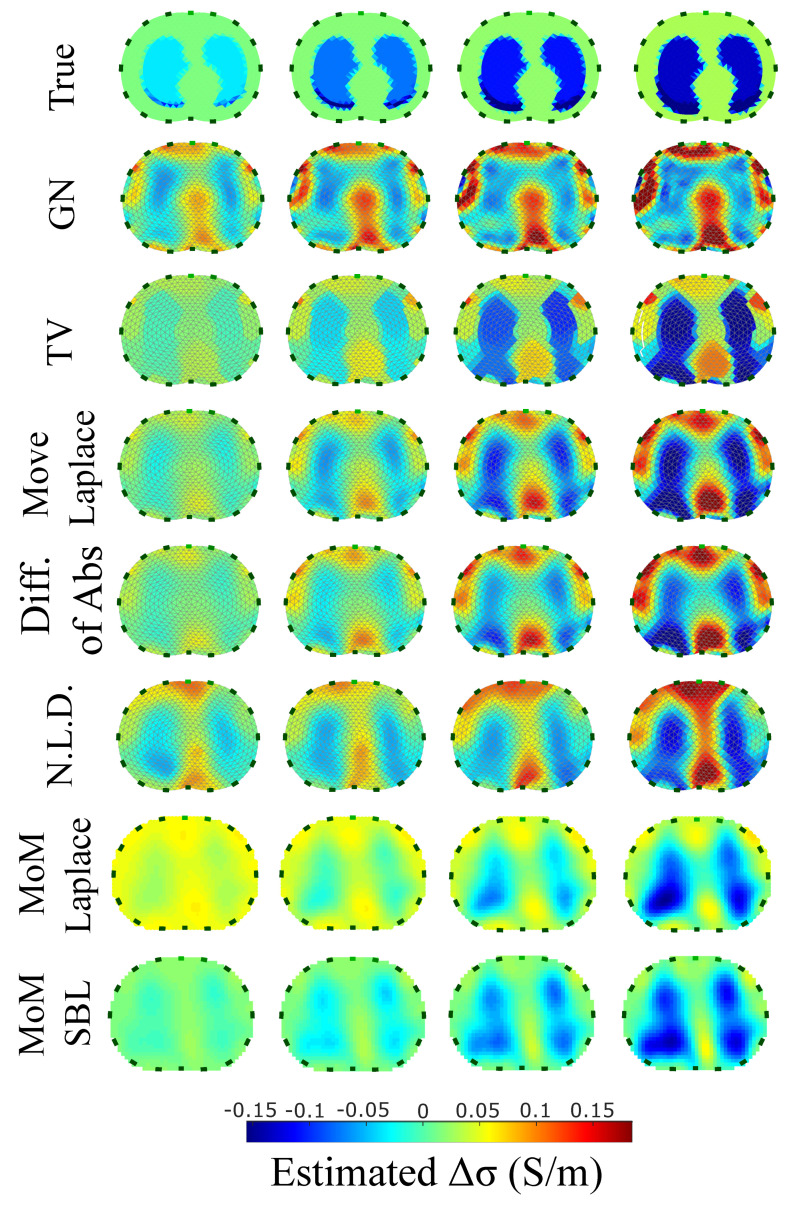
Reconstructed EIT images (conductivity differences) for the first case.

**Figure 10:**
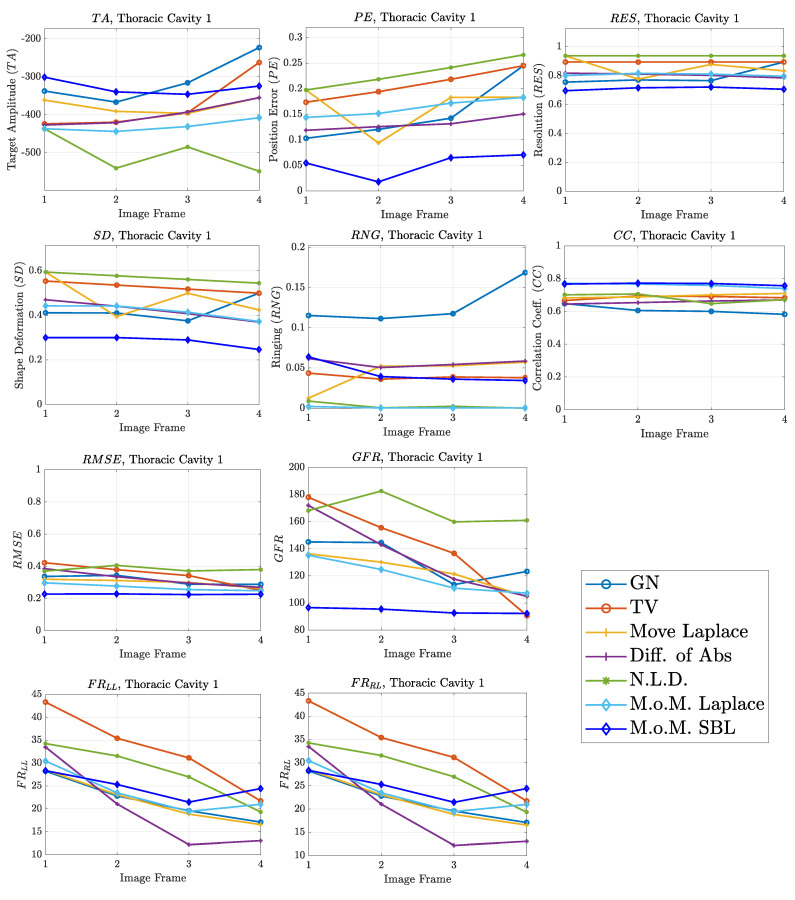
FoM results for the first case.

**Figure 11:**
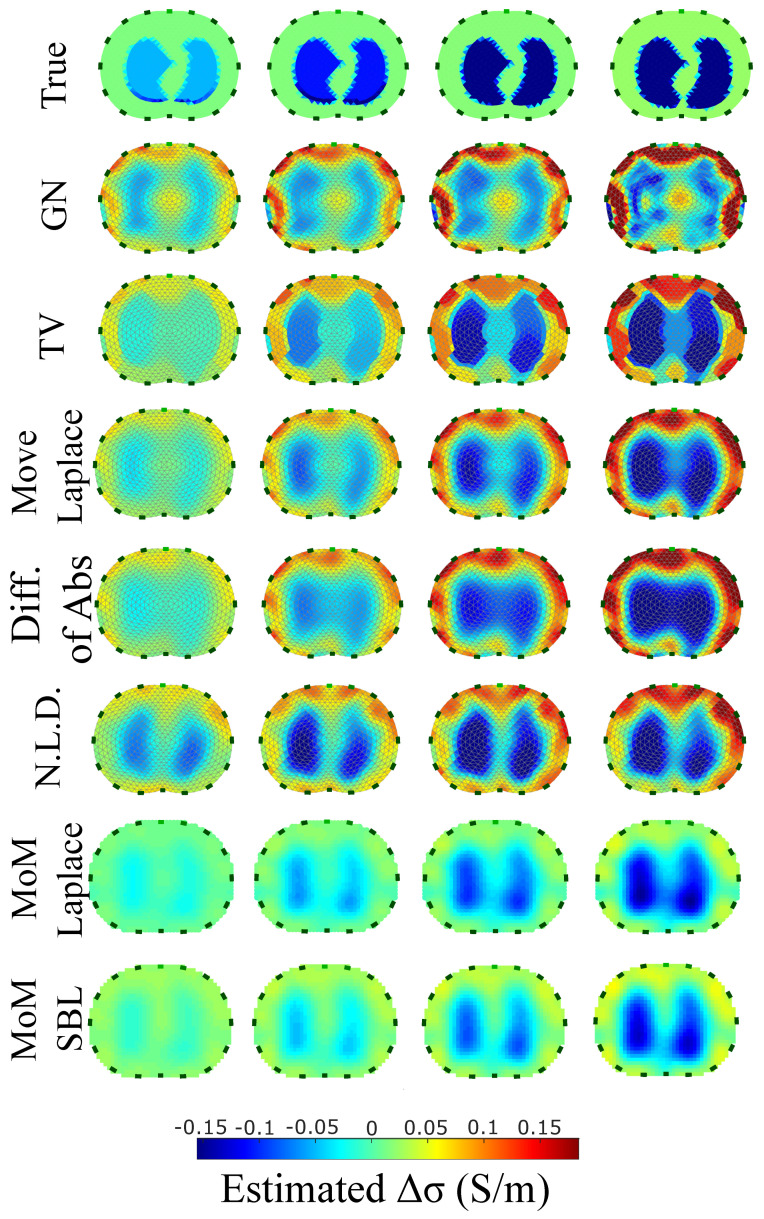
Reconstructed EIT images (conductivity differences) for the second case.

**Figure 12:**
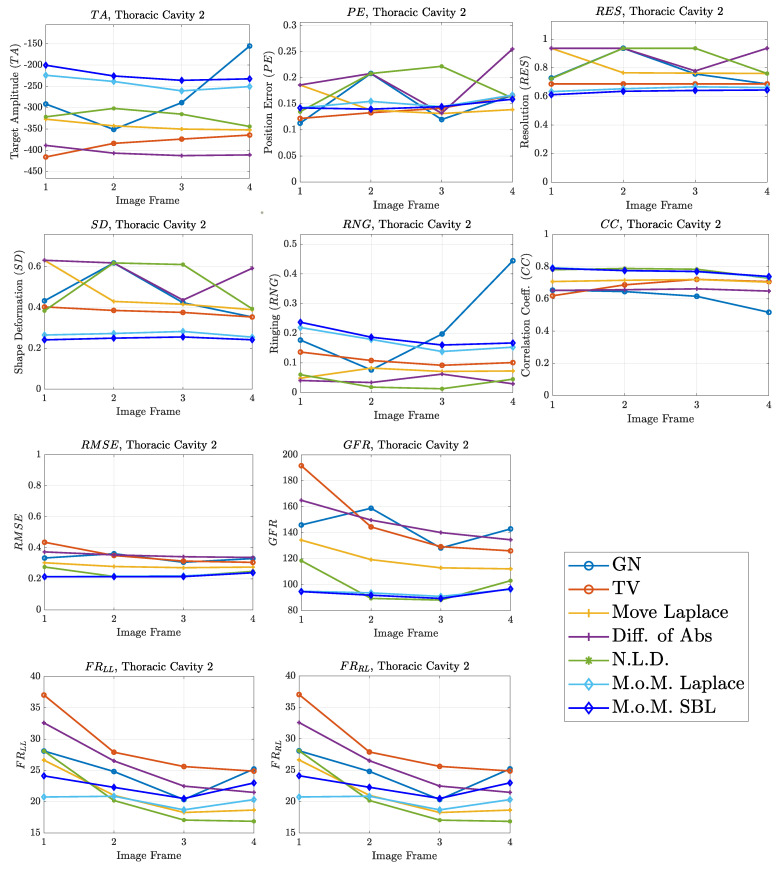
FoM results for the second case.

**Figure 13:**
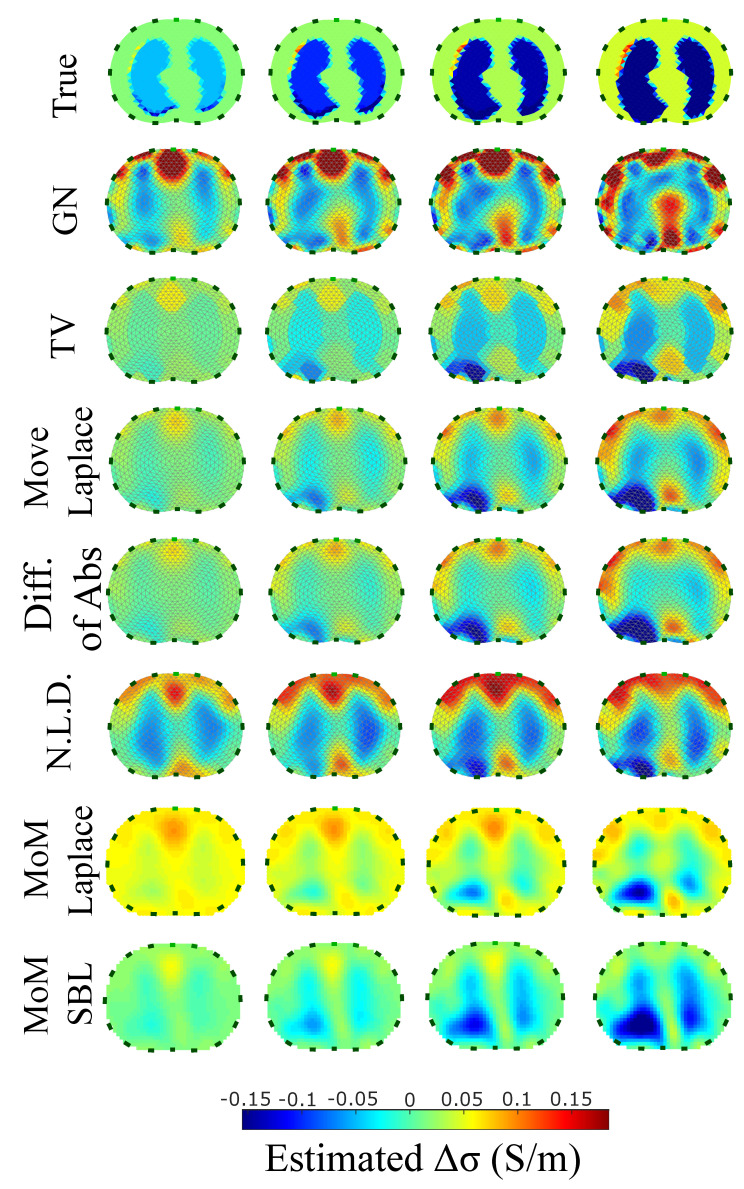
Reconstructed EIT images (conductivity differences) for the third case.

**Figure 14:**
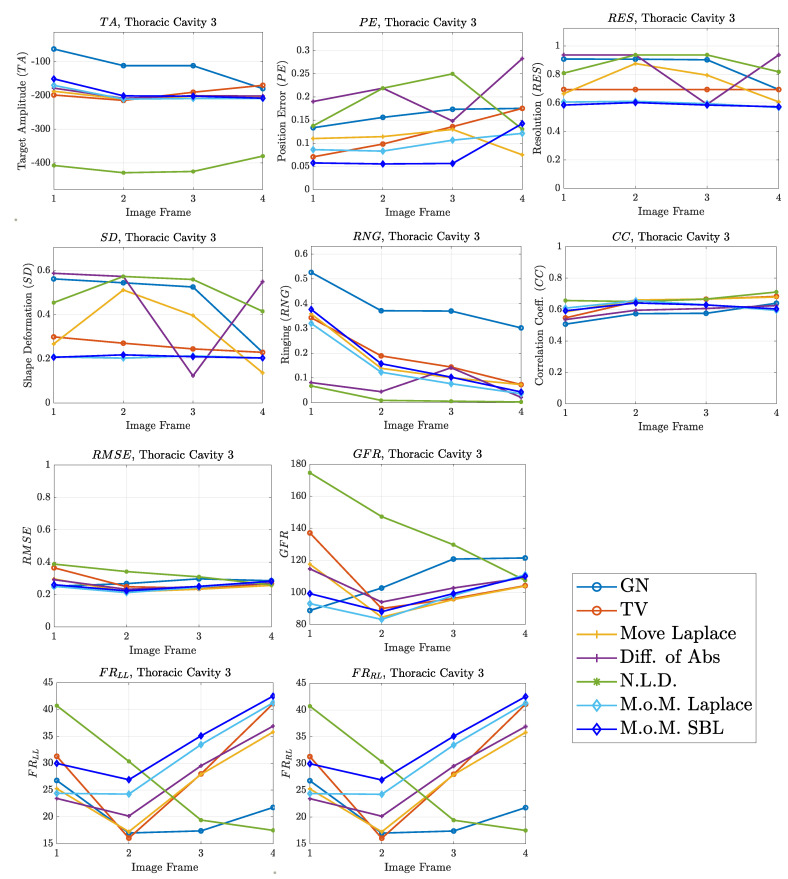
FoM results for the third case.

The resulting image reconstructions and the FoM values demonstrating the simulated cases are depicted in Figure 9, Figure 10, Figure 11, Figure 12, Figure 13 and Figure 14. Specifically, the image reconstructions that represent each one of the structures 1–3 are shown in Figure 9, Figure 11 and Figure 13, respectively. We define the reference image extracted according to the process described in Section 4.3 as the ”true” image. Furthermore, the corresponding FoM values obtained are demonstrated in Figure 10, Figure 12 and Figure 14, respectively.

A visual inspection of the images which resulted from the 1st structure (Figure 9) shows that the air-filled lungs are successfully detected from all the approaches. However, the lungs’ shape and area is deformed, while “pseudo-heart” and boundary artefacts often appear. Such effects are less intense in the MoM Laplace and the MoM SBL cases. As we proceed to the full-inhalation state, the conductivity contrast increases, resulting overall in increased absolute TA, PE and sometimes RNG (Figure 10). At the same time, both local and global FR decrease, while non-significant changes occur at the RES, SD and CC. The RMSE value remains almost constant for all the approaches, except for the TV, where it decreases. Comparing the metric values for each algorithm, better results are obtained by the PM-MoM SBL approach, which shows the lowest and most uniform absolute TA, the lowest PE, RES, SD, RMSE and GFR and the highest CC, followed by the PM-MoM regularization approach.

The images obtained from the simulations of the second structure (Figure 11), which is characterized by closer distance between the lungs, show almost all the artefacts near the boundary instead of between the lungs. The PM-MoM SBL method also shows less intense artefacts, while, along with the regularized PM-MoM and the multiple priors N.L.D. approach, achieving the best CC and lower RMSE, local and GFR values (Figure 12). The proposed method also achieves the lowest absolute and most constant TA, RES and SD. However, the best local FR levels are extracted from the N.L.D, while the PM-MoM shows an increased RNG metric.

The third case results in Figure 13 are characterized by overestimation of the air-related conductivity change near the chest. This occurs due to the presence of lung tissue very close to the chest boundary, as the EIT measurements are sensitive to conductivity changes near the boundary [62]. A visual comparison of the images in Figure 13 indicates that the PM-MoM SBL method has the best performance, an absence of “positive conductivity change” artefacts and less lung deformation. Considering the quantitative results, the PM-MoM SBL approach achieves the lowest PE, RES, SD and RMSE (Figure 14). Although the best TA, RNG, CC and GFR are demonstrated by the GN, N.L.D, N.L.D. and the regularized PM-MoM methods, respectively, the PM-MoM SBL shows the most constant TA, acceptable levels of RNG, a CC which is close to the best one, and the second-lowest GFR.

### 5.2. In Vivo Results

**Figure 15:**
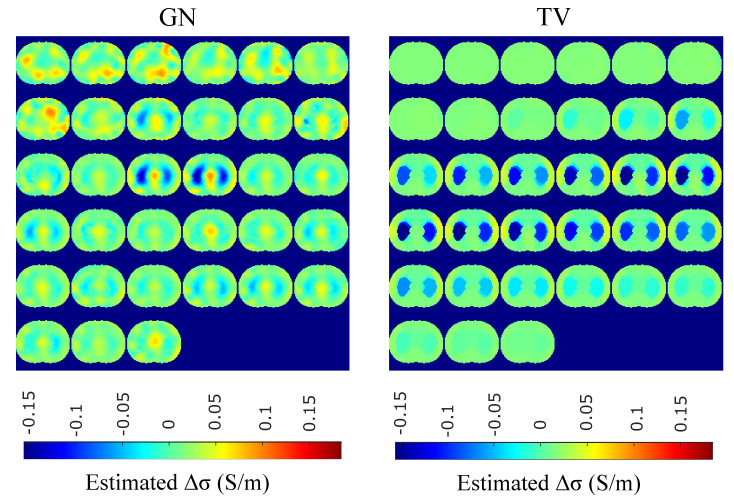
Single–breath in vivo results using the GN and TV approaches.

**Figure 16:**
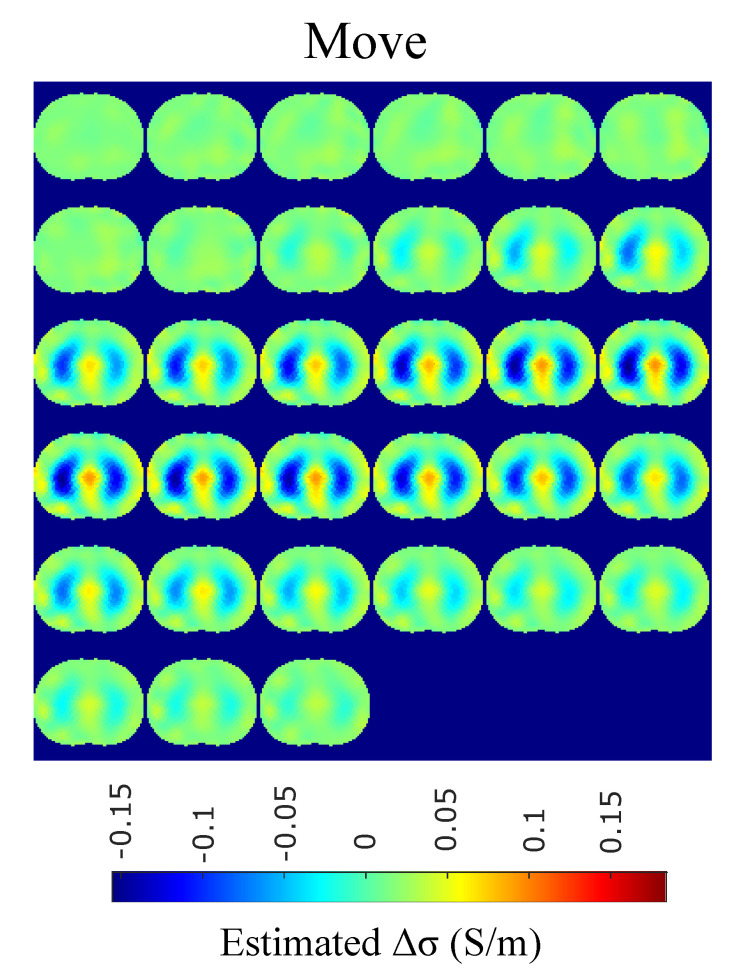
Single–breath in-vivo results using the movement Laplace prior approach.

**Figure 17:**
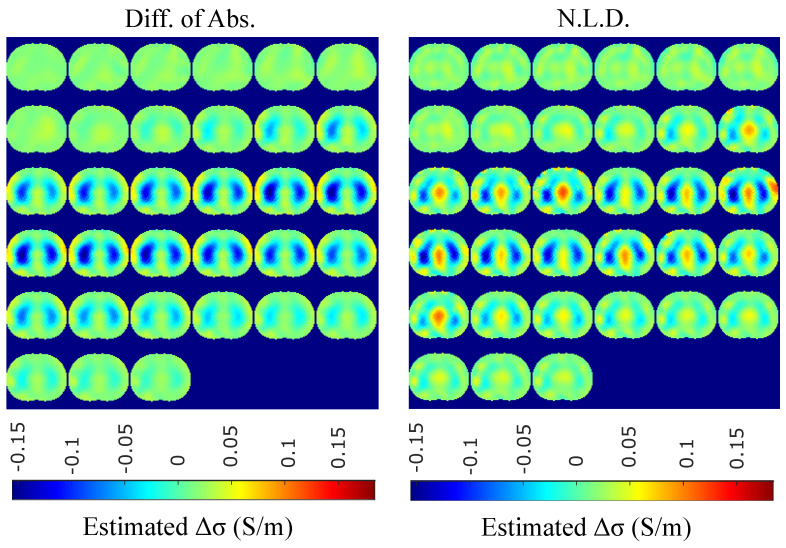
Single–breath in vivo results using the difference of absolute images and multiple priors non-linear difference imaging approaches.

**Figure 18:**
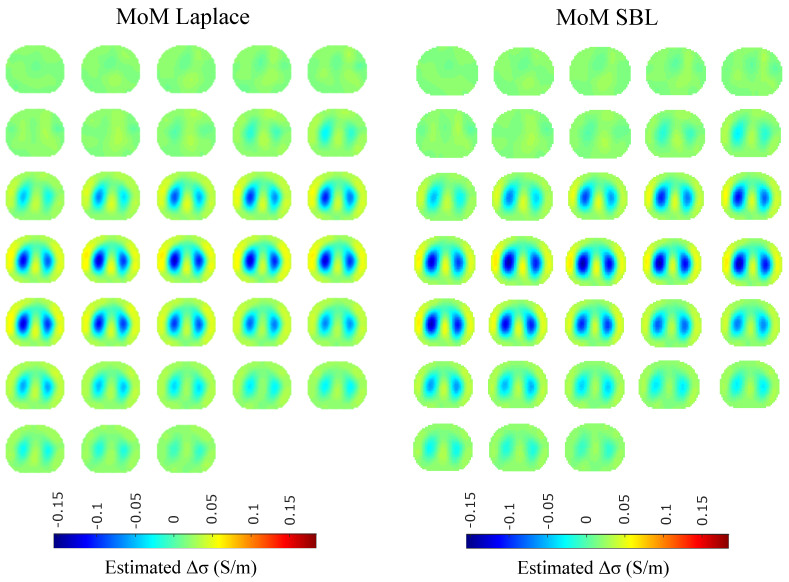
Single–breath in vivo results using the PM-MoM with Laplace regularization prior and the PM-MoM SBL approaches.

The in vivo EIT reconstructed images that demonstrate a subject’s single breath, as described in Section 4.5, are shown in Figure 15, Figure 16, Figure 17 and Figure 18. In particular, Figure 15 shows the reconstructed images using the GN and TV approaches, reviewed in Section 2.4. Figure 16 shows the reconstructed images using the movement prior linear approach, Figure 17 depicts the reconstructed images using the difference of absolute images and the multiple priors non-linear difference imaging (N.L.D.) approaches and Figure 18 demonstrates the regularized and SBL PM-MoM reconstructed images.

A qualitative observation of the images leads to the outcome that all the approaches, except for GN, are able to detect the full-inspiration state. However, the presence of ringing is significant near the centre (between the lungs) in the movement prior, GN and N.L.D. approaches. This might lead to misleading conclusions about the presence of “heart” tissue between the lungs, as mentioned above. In fact, the heart tissue is not directly detectable in difference EIT imaging, since its conductivity does not significantly change during the breath cycle. In addition, any blood-cycle-related conductivity changes are synchronized with the heart rate HR, while the “pseudo-heart” inclusion is synchronized with the breath cycle. Meanwhile, the TV, difference of absolute images and both PM-MoM approaches demonstrate boundary “positive conductivity change” artefacts which are related to the mismatch between the patients’ thoracic shape and ∂Ω. This effect is less intensive in the regularized PM-MoM, PM-MoM SBL, the difference of absolute images and the TV methods, which overall perform better than GN, N.L.D. and movement prior. However, the TV method appears to underestimate the lungs’ area in relation to the total thoracic area. We also observe that the inequality between the lungs’ volumes is successfully detected by most of the approaches (except for TV), but is more clear when enacting PM-MoM (regularized or SBL), difference of absolute images and N.L.D.

### 5.3. Discussion

In this work, a proposed EIT reconstruction method, which combines PM-MoM for the system matrix formulation and SBL for the inverse problem solution, is applied to dynamic thoracic imaging. A number of evaluation criteria is adopted, and an extensive comparison is performed with numerous state-of-the art approaches. Qualitative and quantitative studies have been performed on 3D time-variant non-homogeneous thoracic models. In vivo imaging of a patient’s full-breath cycle has also been applied.

The qualitative results both in the simulation (Figure 9, Figure 11 and Figure 13) and the in vivo studies (Figure 15, Figure 16, Figure 17 and Figure 18) reveal that, overall, the proposed PM-MoM SBL approach shows a better spatial resolution than both the traditional and some more advanced linear and non-linear EIT reconstruction methods, as well as the regularized PM-MoM method. This is confirmed quantitatively in Figure 10, Figure 12 and Figure 14 for the simulated cases. In all three subject-cases, the PM-MoM SBL outperforms the other approaches in most of the FoMs.

It is worthwhile to mention that, of the other approaches, the regularized PM-MoM appears to be the most efficient. In addition, the more recently proposed movement prior, difference of absolute images and multiple priors N.L.D. apaproaches appear to outperform the traditional GN and TV methods.

Considering the time needed for the reconstructions, the best performance (about 50 ms per frame) is achieved by the regularized PM-MoM when using the Laplace $L^{2}$-norm prior with a single step. The movement prior linear approach needs about the same amount of time per reconstruction, while the non-linear GN and TV methods need significantly more time (about 4 to 6 seconds, depending on the number of iterations needed). Due to the relatively high complexity of SBL ($O(N^{2}m^{2}gh)$ per iteration), the PM-MoM SBL approach needs, on average, 5.6 s per image frame reconstruction, when h=4, N=16, m=13, g=1055 and the number of iterations is 5. However, despite the time needed for PM-MoM SBL, the hyperparameter selection process, which is usually time-consuming, is avoided, contrary to the regularization approaches. Finally, the difference of absolute images as well as the multiple priors N.L.D. approaches require significantly longer times to reconstruct the images. The times mentioned above have been achieved using an AMD Ryzen 5 3600 system.

In conclusion, the PM-MoM SBL approach outperforms the regularized MoM one regarding the images’ quality and spatial resolution. Additionally, there is no need for hyperparameter selection, which partially reduces the SBL process complexity effect on execution time. The PM-MoM SBL can be directly applied either for offline imaging (after collecting the measurements) or online on particular breath states where the lung conductivity change is significant. Another choice for faster online imaging is to reduce either the maximum number of iterations $\kappa_{max}$ or tolerance $\epsilon_{min}$ (see Algorithm 1). It is worthwhile to mention that further research on optimizing the SBL approaches’ complexity, as well as evolution in hardware, might improve the total time needed per image reconstruction.

## 6. Conclusions

An EIT reconstruction approach based on a method of moment which expresses the conductivity logarithm with radial basis functions and an SBL method was applied to dynamic EIT lung imaging. In this study, 3D CT-based F.E. thoracic cavities considering 5 breath cycle states and the basic thoracic tissues were developed to simulate the measuring process. Quantitative evaluation was performed using a variety of metrics, and an extensive comparison with other reconstruction approaches took place. In vivo imaging using online available data was also carried out. The results show that the proposed (PM-MoM SBL) approach appears to improve spatial resolution in the reconstructed EIT images. Furthermore, despite the method’s high time complexity, the lack of necessity for hyperparameter selection reduces the effects of this disadvantage. Future research must be performed in the following directions: A) Optimization of the SBL algorithm in terms of imaging quality and time complexity; and B) Application in 3D multi-layer EIT imaging.

## Figures and Tables

**Table 1:** Assigned conductivity and permittivity values to the thoracic models’ tissues for f=100 kHz, according to [47,48,49], and (41)–(42). The admittance is estimated as $\gamma =\sigma +j\omega \epsilon \epsilon_{o}$.

Tissue	σ at 100 kHz (S/m)	$\omega \cdot \epsilon \cdot \epsilon_{o}$ at 100 kHz (F·Hz/m)
Heart	0.215±0.004	0.0548±0.001
Deflated Lung	0.272±0.003	0.029±0.001
Lung State 2	0.225±0.003	0.019±0.001
Lung State 3	0.179±0.003	0.017±0.000
Lung State 4	0.145±0.002	0.029±0.001
Inflated Lung	0.107±0.002	0.014±0.000
Bones	0.021±0.000	0.001±0.000
Skin & Fat	0.045±0.000	0.043±0.000
Muscle (Background)	0.380±0.008	0.024±0.001

**Table 2:** Number of tetrahedral elements and nodes per each 3D thoracic F.E. model.

Model	No of Elements ($L_{e}$)	No of Nodes ($n_{e}$)
Case I, deflated state	133,529	27,328
Case I, state 2	139,486	28,374
Case I, state 3	139,798	28,433
Case I, state 4	142,070	28,814
Case I, inflated state	146,000	29,542
Case II, deflated state	144,329	29,125
Case II, state 2	147,838	29,815
Case II, state 3	146,871	29,688
Case II, state 4	149,887	30,219
Case II, inflated state	150,775	30,359
Case III, deflated state	158,855	31,791
Case III, state 2	158,392	31768
Case III, state 3	159,185	31,937
Case III, state 4	159,550	31,984
Case III, inflated state	160,349	32,159

**Table 3:** Selection of reconstruction parameters per algorithm.

Algorithm	λ	μ	β	*h*
Movement Prior	$8\times 10^{-3}$	1.42	−	−
Gauss–Newton (GN)	$8\times 10^{-3}$	−	−	−
Total Variation (TV)	$10^{-6}$	−	$10^{-3}$	−
Difference of Absolute Images	$5\times 10^{-2}$	−	−	−
Multiple Priors (N.L.D.)	$\lambda_{1}:5\times 10^{-5}$ $\lambda_{ROI}:8\times 10^{-5}$	−	$10^{-3}$	−
PM-MoM Laplace	0.2	−	−	−
PM-MoM SBL	−	−	−	4

## Data Availability

The data used in this study are openly available in Public Lung Database to address drug response (PLD) at 10.1109/IEMBS.2009.5334807, reference number [44].
